# Transcriptomic and microRNA Expression Profiles Identify Biomarkers for Predicting Neo-Chemoradiotherapy Response in Esophageal Squamous Cell Carcinomas (ESCC)

**DOI:** 10.3389/fphar.2021.626972

**Published:** 2021-04-15

**Authors:** Jian Wang, Pengyi Yu, Judong Luo, Zhiqiang Sun, Jingping Yu, Jianlin Wang

**Affiliations:** ^1^ Department of Radiotherapy, Jiangyin People’s Hospital, Jiangyin, China; ^2^ Department of Cardiothoracic Surgery, The Third Affiliated Hospital of Soochow University, Jiangsu, China; ^3^ Department of Radiotherapy, The Affiliated Changzhou Second People's Hospital of Nanjing Medical University, Jiangsu, China

**Keywords:** esophageal neoplasms, differentially expressed genes, prognosis, MMP12, neo-chemoradiotherapy

## Abstract

Neo-chemoradiotherapy (nCRT) before surgery is a standard treatment for locally advanced esophageal cancers. However, the treatment outcome of nCRT varied with different patients. This study aimed to identify potential biomarkers for prediction of nCRT-response in patients with esophageal squamous cell carcinoma (ESCC). Microarray datasets of nCRT responder and non-responder samples (access number GSE45670 and GSE59974) of patients with ESCC were downloaded from Gene Expression Omnibus (GEO) database. The mRNA expression profiles of cancer biopsies from four ESCC patients were analyzed before and after nCRT. Differentially expressed genes (DEGs) and miRNAs were screened between nCRT responder and non-responder ESCC samples. Functional enrichment analysis was conducted for these DEGs followed by construction of protein-protein interaction (PPI) network and miRNA-mRNA regulatory network. Finally, univariate survival analysis was performed to identify candidate biomarkers with prognostic values in ESCC. We identified numerous DEGs and differentially expressed miRNAs from nCRT responder group. GO and KEGG analysis showed that the dysregulated genes were mainly involved in biological processes and pathways, including “response to stimulus”, “cellular response to organic substance”, “regulation of signal transduction”, “AGE-RAGE signaling pathway in diabetic complications”, and “steroid hormone biosynthesis”. After integration of PPI network and miRNA-mRNA network analysis, we found eight genes, TNF, AKR1C1, AKR1C2, ICAM1, GPR68, GNB4, SERPINE1 and MMP12, could be candidate genes associated with disease progression. Univariate cox regression analysis showed that there was no significant correlation between dysregulated miRNAs (such as hsa-miR-34b-3p, hsa-miR-127-5p, hsa-miR-144-3p, and hsa-miR-486-5p, et al.) and overall survival of ESCC patients. Moreover, abnormal expression of MMP12 was significantly correlated with pathological degree, TNM stage, lymph nodes metastasis, and overall survival of ESCC patients (*p* < 0.05). Taken together, our study identified that MMP12 might be a useful tumor biomarker and therapeutic target for ESCC.

## Introduction

Esophageal squamous cell carcinoma (ESCC) is a primary histological type of esophageal cancer worldwide. In the United States, approximately 18,440 new cases were diagnosed and 13,100 deaths occurred in 2020 (Siegel et al., 2020). China is one of the areas with highest incidence of esophageal cancer. According to the cancer statistics in China, an estimate of 477,900 newly diagnosed esophagus carcinoma cases were found in China, and 375,000 individuals died of this disease (Chen et al., 2016). Over the past decades, the incidence of esophageal cancer has been decreased slightly in the United States, whereas most cases of this disease occurred in developing countries. ESCCs in China bear more than half of global burden (Bray et al., 2018; He et al., 2019).

Esophageal cancer has a poor prognosis when it is diagnosed at advanced stage. The 5 years survival rate would decrease to 4% when metastasis occurred (Shaheen et al., 2017). As for locally advanced ESCC, neo-chemoradiotherapy (nCRT) before surgery is the primary management. However, patients always suffer from disease recurrence after resection. Also, the outcome of nCRT varied among patients. Patients who response to nCRT acquire superior survival time while those with no response suffer a poor prognosis (Berger et al., 2005). Besides, nCRT may increase postoperative complications, and clinical parameters (TNM classification, tumor location) could not predict response to nCRT (Rohatgi et al., 2005; Wen et al., 2014). Therefore, identification of novel biomarkers that could predict response to nCRT would be beneficial for ESCC management. In addition, it would help clinical doctors to discontinue non-effective treatments, and thereby avoid overtreatment for non-responders.

**FIGURE 1:**
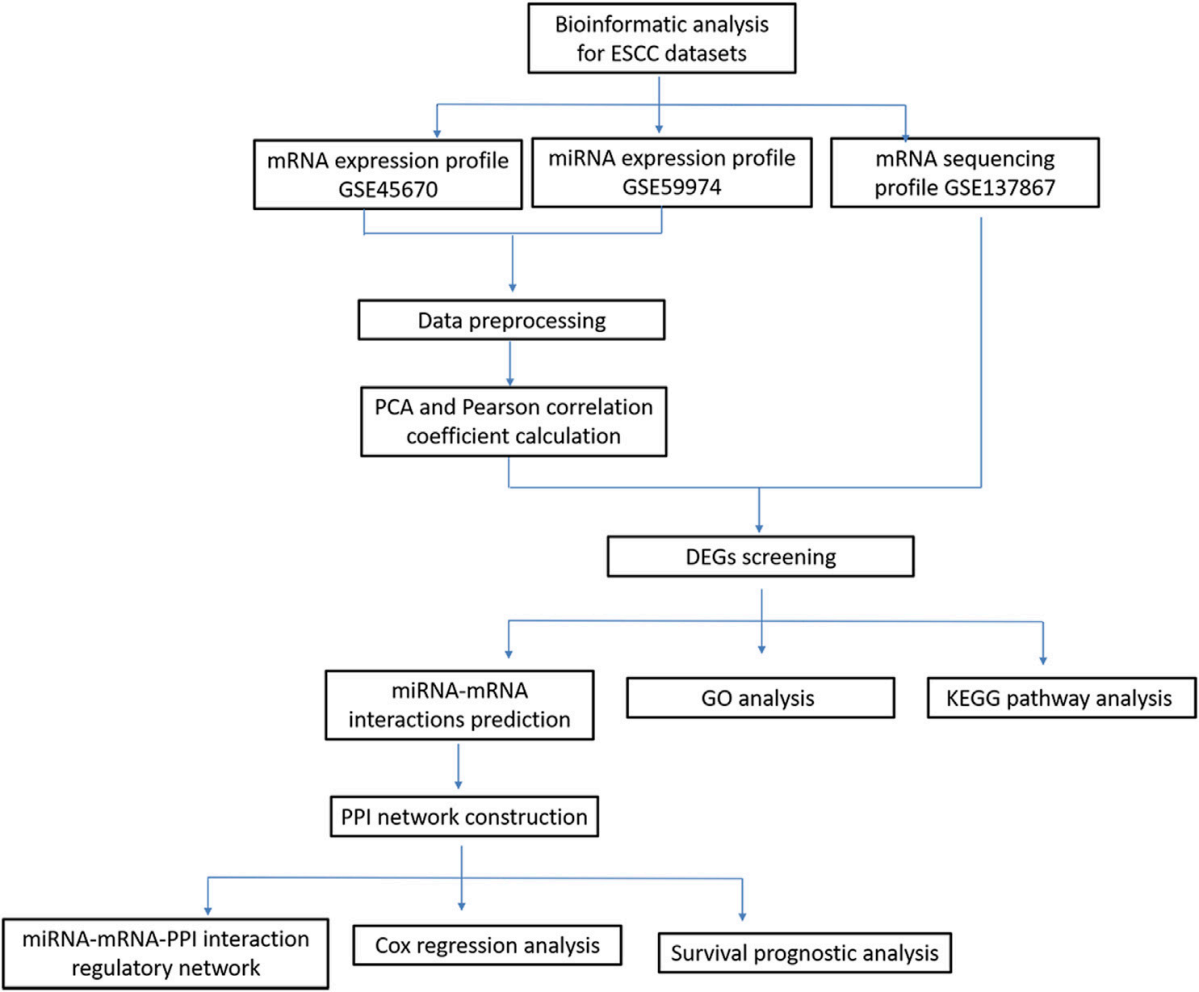
A schematic diagram of bioinformatics analysis for ESCC datasets.

In recent years, the development of microarray analysis and RNA sequencing technology provided favorable information for exploring the association of gene expression and clinical outcomes (Meyerson et al., 2010; Ross and Cronin, 2011). In this study, based on RNA sequencing and bioinformatics analysis, we identified differentially expressed genes (DEGs) or differentially expressed miRNA between nCRT responders and non-responders in patients with ESCC. Additionally, protein-protein interaction (PPI) network integrated with miRNA-mRNA network were constructed to explore the candidate genes related to disease progression. Cox regression analysis was finally conducted to investigate the correlations between survival times and candidate genes or miRNAs in ESCC patients. A schematic diagram of bioinformatics analysis for ESCC datasets were shown in Figure 1. Our study investigated the transcriptomic expression patterns of ESCC, and identified novel biomarkers with prognostic values that could provide a better understanding of ESCC progression.

## Materials and Methods

### Data Source and Data Processing

The gene expression profile GSE45670 (Wen et al., 2014) and miRNA expression profile GSE59974 (Fu et al., 2016) were downloaded from the Gene Expression Omnibus (GEO) database. The two datasets were derived from the same clinical samples that consist of 17 treatment non-responders and 11 responders. GSE45670 dataset were tested on platform of hg-u133_plus_2 Affymetrix Human Genome U133 Plus 2.0 and GSE45670 was analyzed on Agilent-038169 human miRNA novirus v18.0 platform.

In addition, cancer biopsies were taken from four patients before and after nCRT in Changzhou No.2 People’s Hospital, the Affiliated Hospital of Nanjing Medical University. This study was approved by the Ethical Research Committee of hospital. Transcriptomics data (under access number GSE137867, https://www.ncbi.nlm.nih.gov/geo/query/acc.cgi?acc=GSE137867) were obtained by RNA sequencing technology and mRNA expression was analyzed for these clinical specimens. GSE137867 were considered as validation datasets. DEGs were screened from the three datasets and overlapped genes were considered as candidate genes related to ESCC.

Data normalization was performed for these microarray data. We extracted the gene expression values from series matrix files, and converted corresponding probe ID into gene symbols. After removal of abnormal information, we selected the expressed values from ESCC responders or non-responders to conduct Principal Components Analysis (PCA).

### PCA Analysis and DEGs Screening

PCA is a multivariate technique that converted multiple correlated variables into cohort of values linearly, and uncorrelated variables represented principal components. It has been extensively used to explore high-dimensional data, such as genomic and transcriptomic expression data (Ringnér, 2008; Ellsworth et al., 2017). In this study, we used PCA method to investigate distribution of samples between the experimental group and control groups. After detection and removal of abnormal samples, we finally obtained a series of specimen with high similarity.

The miRNA matrix and mRNA expression profiles were normalized based on quantile normalization method. Limma package (Ritchie et al., 2015) was used to screen DEGs and miRNAs between experimental group and normal control group by setting thresholds as *p* < 0.1 and fold change >1.5 (|log2FC| > 0.585). Moreover, with the same cutoff criteria, we screened the overlapped DEGs of GSE137867 and GEO datasets to conduct further analysis.

### Functional Enrichment Analysis for DEGs Associated with ESCC

Gene Ontology (GO) (Ashburner et al., 2000) and Kyoto Encyclopedia of Genes and Genomes (KEGG) (Ogata et al., 2000) signaling pathway enrichment analysis were conducted for DEGs. Using fisher's exact test, we screened a cohort of biological processes and pathway categories enriched by DEGs. *p* < 0.05 was considered as significant difference, and the column with smaller *p* value represented a closer association between DEGs and pathway categories.

### Prediction of miRNA-mRNA Interactions

We further predicted the miRNA-mRNA interactions by examining various databases [TargetScan (Agarwal et al., 2015), miRTarBase (Chih-Hung et al., 2017), miRDB (Nathan and Wang, 2014) and miRanda (Doron et al., 2008)]. As for the upregulated miRNAs, we explored their correlations with down-regulated target genes. Subsequently, we focused on the downregulated miRNAs, and investigated their correlations with upregulated target genes in nCRT responders. Cytoscape software was used to visualize the relationships of differentially expressed miRNAs and mRNAs.

### Construction of Regulatory Network

The online tool STRING (https://string-db.org/) (Christian et al., 2003) was used to analyze the interactions of proteins. Interaction score >0.7 (high confidence) was set as the cut-off criteria. After screening the correlation pairs, Cytoscape (Shannon et al., 2003) was used to analyze the topological properties (connectivity degree, closeness degree and betweenness degree) of PPI network. The proteins with high scores were considered as hub factors in network and might be key candidate genes in disease progression.

In addition, we integrated the PPI network and miRNA-mRNA regulatory network by using Cytoscape software to predict the candidate miRNAs involved in ESCC progression.

### Survival Analysis

Survival package (Cox, 1972) in R software was used to construct cox regression model. Univariate cox regression analysis and survival analysis were performed to identify crucial genes and miRNAs related to ESCC survival based on the TCGA datasets.

## Results

### Screening DEGs From nCRT Responders of ESCCs

PCA analysis and correlation analysis on samples were conducted for mRNA expression dataset GSE45670. Under the threshold of *p* < 0.1 and fold change >1.5 (|log2FC| > 0.585), we screened a total of 1311 DEGs from nCRT responders and non-responders. We found 672 upregulated and 639 down-regulated genes. The same cutoff criteria were applied for RNA-Seq profile GSE137867, and numerous DEGs were screened in specimens before and after nCRT.

**FIGURE 2:**
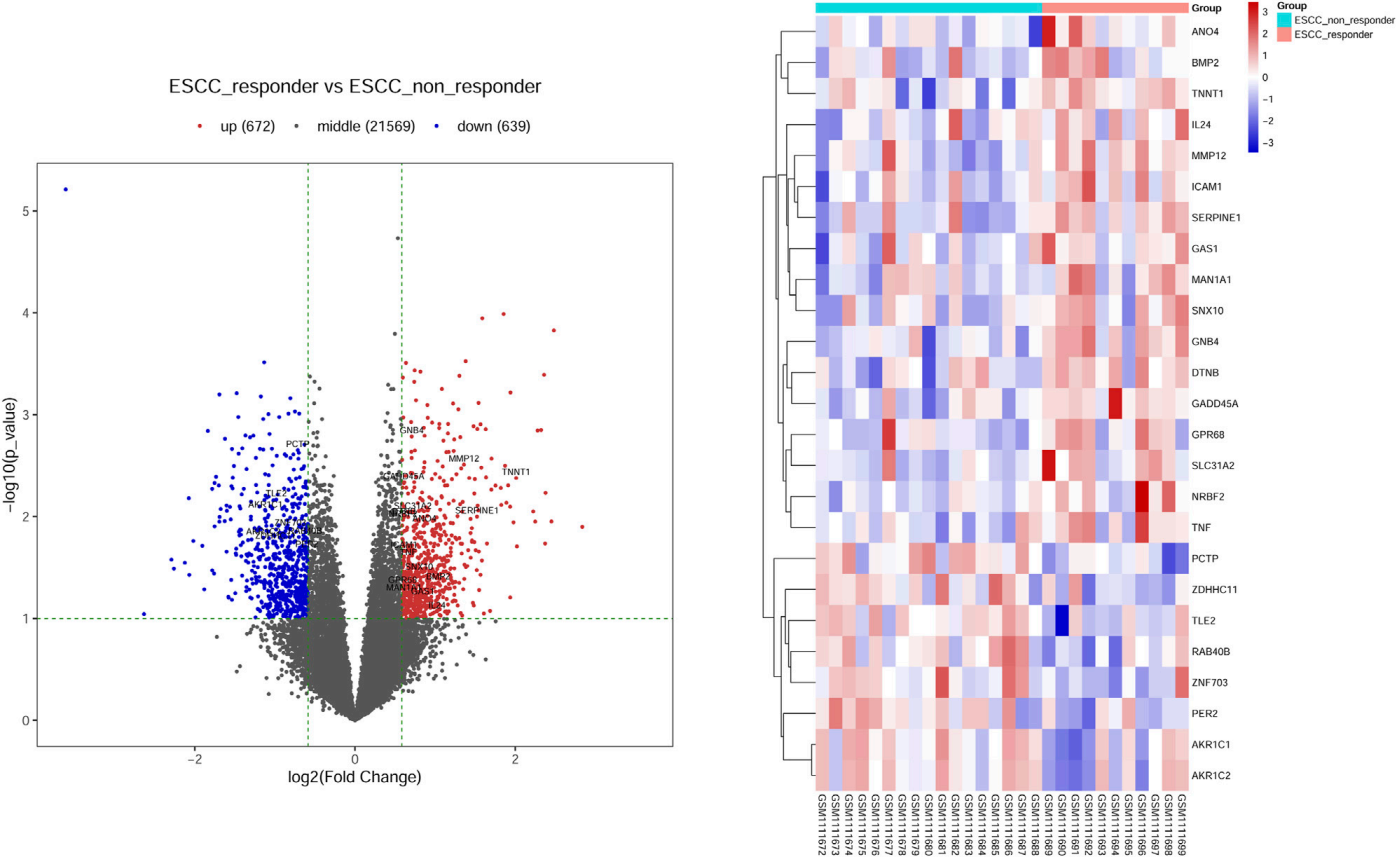
Screening the differential expression genes (DEGs) in ESCC-nCRT responder and non-responder samples. **(A)** Volcano plot visualized the distribution of DEGs. Red dots represented up-regulated genes and blue dots were down-regulated genes. The overlapped genes were named in figures represented the up-regulated or down-regulated genes in both datasets. **(B)** Bidirectional clustering analysis of DEG in nCRT responder and non-responder samples. These genes are overlapped genes dysregulated in both datasets. The color changed from blue to red represented the expression level from low to high.

**FIGURE 3:**
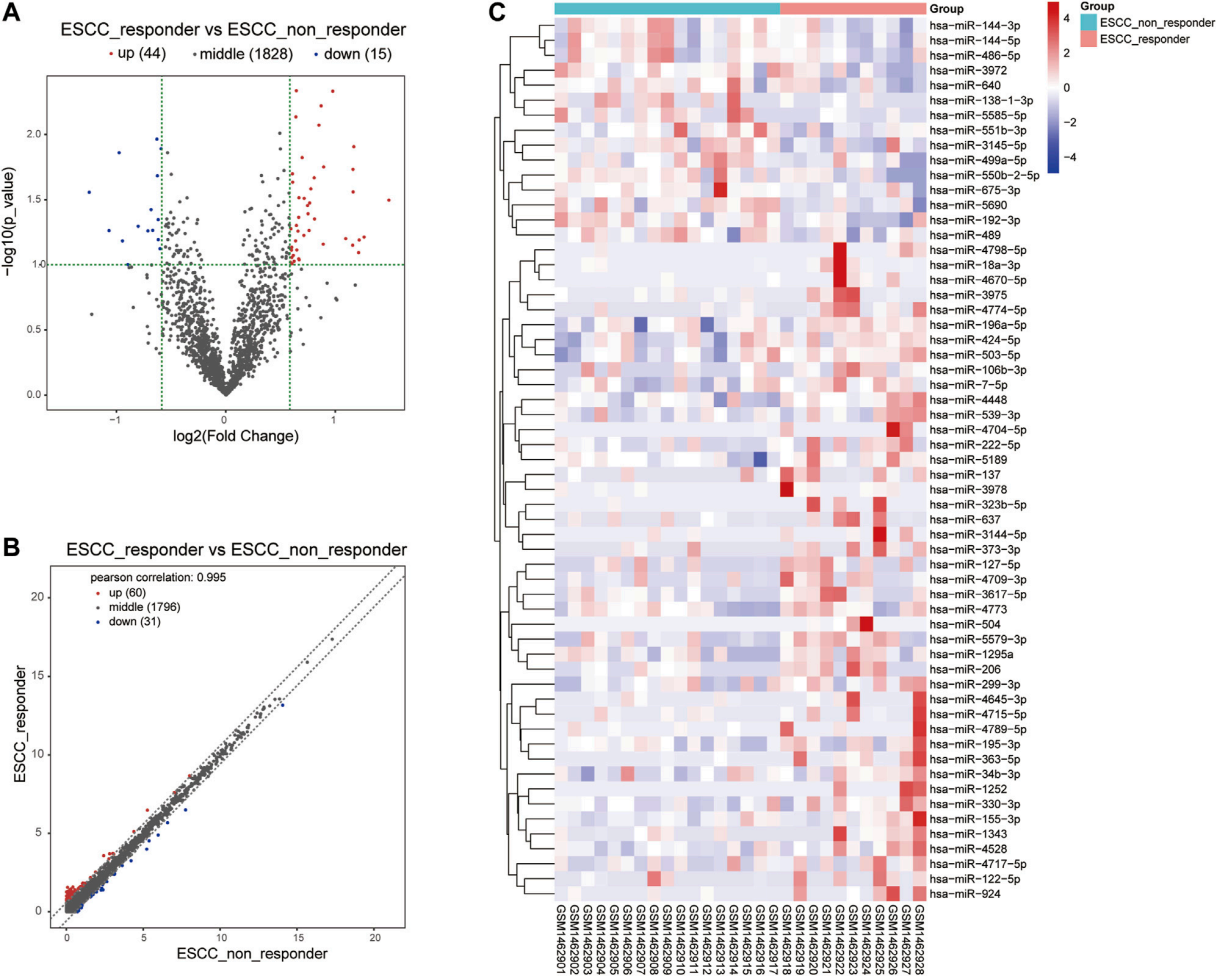
Volcano plot and heat map visualized the differentially expressed miRNA screening between nCRT responders and non-responders. **(A, B)** Scatter plot and Volcano plot represented the differentially expressed miRNAs found by correlation analysis of ESCC samples. **(C)** Clustering analysis of differentially expressed miRNAs between nCRT responder and non-responder samples. The color changed from blue to red represented the expression level from low to high.

Moreover, we integrated the overlapped DEGs between RNA-Seq profile and GEO datasets. Of these gene overlaps, 17 genes were both upregulated in two datasets while eight genes were both downregulated in two datasets (Table 1). Volcano plot and bidirectional clustering analysis were performed for these DEGs, and results were shown in Figure 2. Furthermore, the differentially expressed miRNAs were identified by setting the thresholds of *P* < 0.1 and fold change >1.5 (|log2FC| > 0.585). Scatter plot and volcano plot were used to visualize the results. Finally, we identified 44 upregulated miRNAs and 15 downregulated miRNAs from the nCRT responders compared with non-responders. The differentially expressed miRNAs are listed in Table 2 and heatmaps are visualized in Figure 3.

**TABLE 1:** Twenty five DEGs were identified from two mRNA profiles (GSE45670 dataset and GSE137867 dataset), including 17 upregulated genes and 8 down-regulated genes in the ESCC responder groups compared with control non-responder groups.

DEGs	Gene names
Up-regulated	ANO4, BMP2, DTNB, GADD45A, GAS1, GNB4, GPR68, ICAM1, IL24, MAN1A1, MMP12, NRBF2, SERPINE1, SLC31A2, SNX10, TNF, TNNT1
Down-regulated	AKR1C1, AKR1C2, PCTP, PER2, RAB40B, TLE2, ZDHHC11, ZNF703

**TABLE 2:** Fifety nine differential expressed miRNAs were identified from miRNA profiles (GSE59974 dataset), including 44 upregulated genes and 15 down-regulated genes in the ESCC responder groups compared with control groups.

miRNAs	Gene names
Up-regulated	hsa-miR-106b-3p, hsa-miR-122-5p, hsa-miR-1252, hsa-miR-127-5p, hsa-miR-1295a, hsa-miR-1343, hsa-miR-137, hsa-miR-155-3p, hsa-miR-18a-3p, hsa-miR-195-3p, hsa-miR-196a-5p, hsa-miR-206, hsa-miR-222-5p, hsa-miR-299-3p, hsa-miR-3144-5p, hsa-miR-323b-5p, hsa-miR-330-3p, hsa-miR-34b-3p, hsa-miR-3617-5p, hsa-miR-363-5p, hsa-miR-373-3p, hsa-miR-3975, hsa-miR-3978, hsa-miR-424-5p, hsa-miR-4448, hsa-miR-4528, hsa-miR-4645-3p, hsa-miR-4670-5p, hsa-miR-4704-5p, hsa-miR-4709-3p, hsa-miR-4715-5p, hsa-miR-4717-5p, hsa-miR-4773, hsa-miR-4774-5p, hsa-miR-4789-5p, hsa-miR-4798-5p, hsa-miR-503-5p, hsa-miR-504, hsa-miR-5189, hsa-miR-539-3p, hsa-miR-5579-3p, hsa-miR-637, hsa-miR-7-5p, hsa-miR-924
Down-regulated	hsa-miR-138-1-3p, hsa-miR-144-3p, hsa-miR-144-5p, hsa-miR-192-3p, hsa-miR-3145-5p, hsa-miR-3972, hsa-miR-486-5p, hsa-miR-489, hsa-miR-499a-5p, hsa-miR-550b-2-5p, hsa-miR-551b-3p, hsa-miR-5585-5p, hsa-miR-5690, hsa-miR-640, hsa-miR-675-3p

### GO and KEGG Analysis for These Candidate Genes

The GO analysis results revealed that these dysregulated DEGs were mainly associated with several biological processes (Table 3), such as “response to stimulus” (count = 18, FDR = 0.001617666), “regulation of signaling” (count = 12, FDR = 0.001658521), “response to organic substance” (count = 11, FDR = 0.001658521), “cellular response to organic substance” (count = 11, FDR = 0.001658521), and “regulation of signal transduction” (count = 11, FDR = 0.002048231). The KEGG pathways (Table 4) enriched by these genes were “AGE-RAGE signaling pathway in diabetic complications” (count = 3, p = 5.23e−04), “Epstein-Barr virus infection” (count = 3, p = 3.91e−03), “steroid hormone biosynthesis” (count = 2, p = 3.91e−03), “African trypanosomiasis” (count = 2, p = 1.63e−03) and “malaria” (count = 2, *p* = 2.85e−03).

**TABLE 3:** Gene Ontology analysis of differentially expressed genes associated with ESCC (Top ten biological process terms).

Category	Description	Count	*p* value	FDR
BP	GO:0071396∼cellular_response_to_lipid	6	2.80e−06	0.001617666
BP	GO:0050896∼response_to_stimulus	18	2.84e−06	0.001617666
BP	GO:0071395∼cellular_response_to_jasmonic_acid_stimulus	2	3.63e−06	0.001617666
BP	GO:0023051∼regulation_of_signaling	12	6.06e−06	0.001658521
BP	GO:0010033∼response_to_organic_substance	11	7.33e−06	0.001658521
BP	GO:0071310∼cellular_response_to_organic_substance	10	7.44e−06	0.001658521
BP	GO:0009966∼regulation_of_signal_transduction	11	1.32e−05	0.002048231
BP	GO:0071222∼cellular_response_to_lipopolysaccharide	4	1.46e−05	0.002048231
BP	GO:0030155∼regulation_of_cell_adhesion	6	1.46−-05	0.002048231
BP	GO:2000351∼regulation_of_endothelial_cell_apoptotic_process	3	1.54e−05	0.002048231

Category stands for GO terms and BP refers to biological process.

**TABLE 4:** KEGG pathway analysis of differentially expressed genes in ESCC.

Category	Description	Count	*p* value	FDR	Genes
KEGG	hsa04933∼AGE-RAGE_signaling_pathway_in_diabetic_complications Homo_sapiens_(human)	3	5.23e−04	0.058021807	ICAM1/SERPINE1/TNF
KEGG	hsa05143∼African_trypanosomiasis Homo_sapiens_(human)	2	1.63e−03	0.08638883	ICAM1/TNF
KEGG	hsa05144∼Malaria Homo_sapiens_(human)	2	2.85e−03	0.08638883	ICAM1/TNF
KEGG	hsa05169∼Epstein-Barr_virus_infection Homo_sapiens_(human)	3	3.91e−03	0.08638883	GADD45A/ICAM1/TNF
KEGG	hsa00140∼Steroid_hormone_biosynthesis Homo_sapiens_(human)	2	4.24e−03	0.08638883	AKR1C1/AKR1C2
KEGG	hsa05217∼Basal_cell_carcinoma Homo_sapiens_(human)	2	4.67e−03	0.08638883	BMP2/GADD45A
KEGG	hsa04115∼p53_signaling_pathway Homo_sapiens_(human)	2	6.06e−03	0.095935394	GADD45A/SERPINE1
KEGG	hsa05323∼Rheumatoid_arthritis Homo_sapiens_(human)	2	9.54e−03	0.095935394	ICAM1/TNF
KEGG	hsa04350∼TGF-beta_signaling_pathway Homo_sapiens_(human)	2	0.01	0.095935394	BMP2/TNF
KEGG	hsa04713∼Circadian_entrainment Homo_sapiens_(human)	2	0.01	0.095935394	GNB4/PER2

### PPI Network Analysis and Prediction of miRNA-Gene Interactions

**FIGURE 4:**
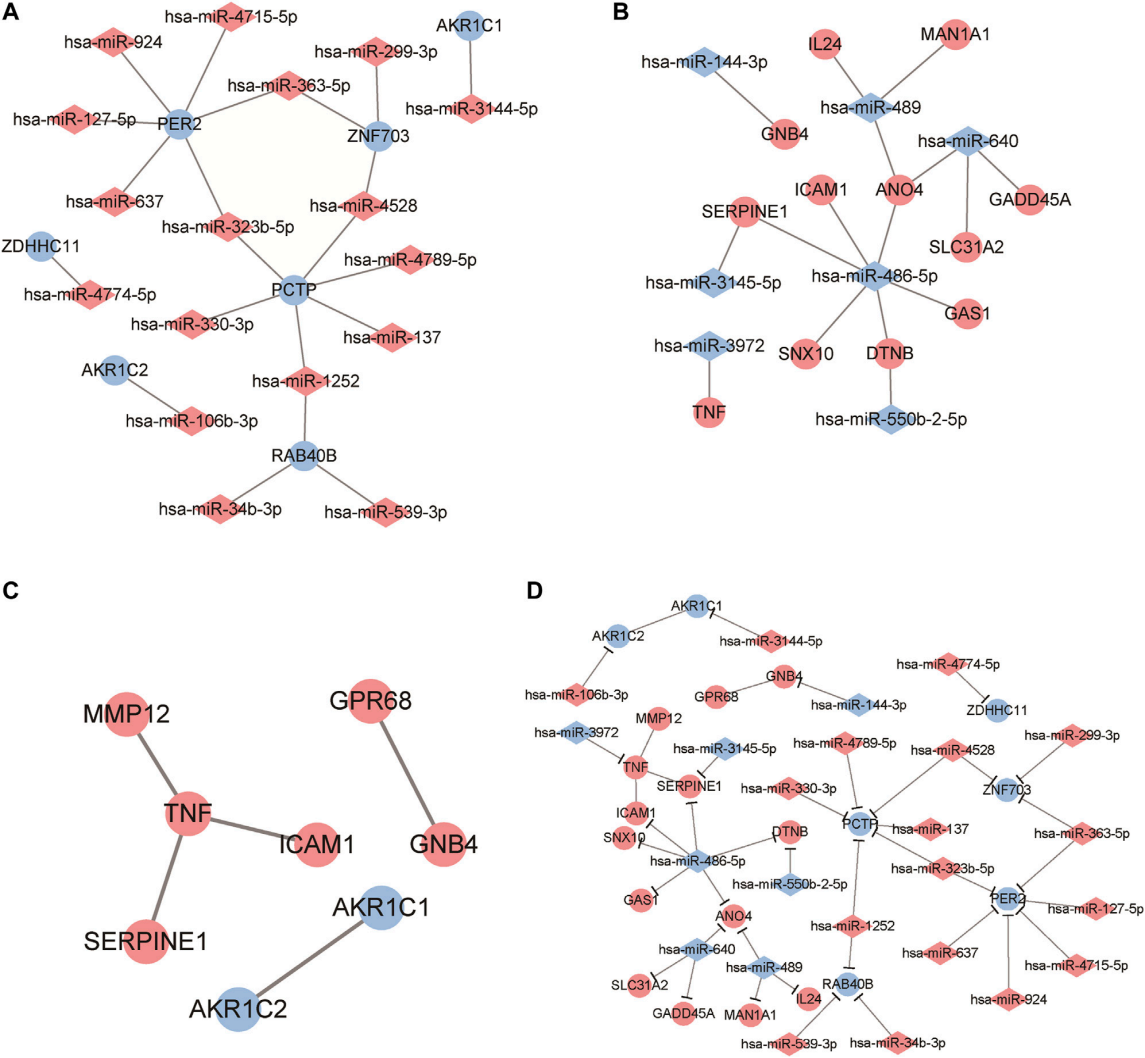
Identification of candidate genes based on protein-protein interaction (PPI) network analysis and miRNA-mRNA regulatory network construction. **(A, B)** Regulatory network visualized the correlation of dysregulated miRNA and mRNAs in ESCC samples. The circles represent mRNA while diamonds refer to miRNAs. Red mean to up-regulated genes and blue refer to down-regulated gene. **(C)** PPI network analysis for these differentially expressed genes. A red dot represents an up-regulated gene and a blue dot is a down-regulated gene. **(D)** The miRNA-mRNA regulatory network analysis to identify crucial genes related to ESCC progression. Red colors represent up-regulated while blue colors refer to down-regulated genes. Diamond and circles represent miRNA and mRNA.

By predicting the target genes of the upregulated miRNAs and downregulated miRNAs, we built two miRNA-target networks for upregulated miRNAs (Figure 4A) and downregulated miRNAs (Figure 4B). In the regulatory networks, several molecules were identified as hub genes for high degree of connectivity, including PER2 (degree = 6), PCTP (degree = 6), and hsa-miR-486-5p (degree = 6).

Using the online tool STRING and Cytoscape software, we constructed a PPI network for DEGs (Table 5; Figure 4C) and obtained five interaction pairs among eight genes, including tumor necrosis factor (TNF), Aldo-keto reductase family one member C1 (AKR1C1), AKR1C2, intercellular adhesion molecule 1 (ICAM1), ovarian cancer G-protein coupled receptor 1 (GPR68), guanine nucleotide-binding protein subunit beta-4 (GNB4), plasminogen activator inhibitor-1 (SERPINE1) and matrix metalloproteinase-12 (MMP12).

**TABLE 5:** Protein-protein interaction network analysis for differential expressed genes in ESCC samples based on connectivity degrees evaluation.

Gene	Degree
TNF	3
AKR1C1	1
AKR1C2	1
ICAM1	1
GPR68	1
GNB4	1
SERPINE1	1
MMP12	1

Only AKR1C1 and AKR1C2 were down-regulated genes while other genes were upregulated.

By integrating PPI network and miRNA-mRNA network, we finally constructed a regulatory network that consisted with miRNAs and genes. Among these genes, PER2, PCTP, TNF and hsa-miR-486-5p exhibited higher interactions than other genes. Thus, these genes were identified as hub genes related to ESCC progression.

### Identifying Crucial Genes with Prognostic Values in nCRT Responders

**FIGURE 5:**
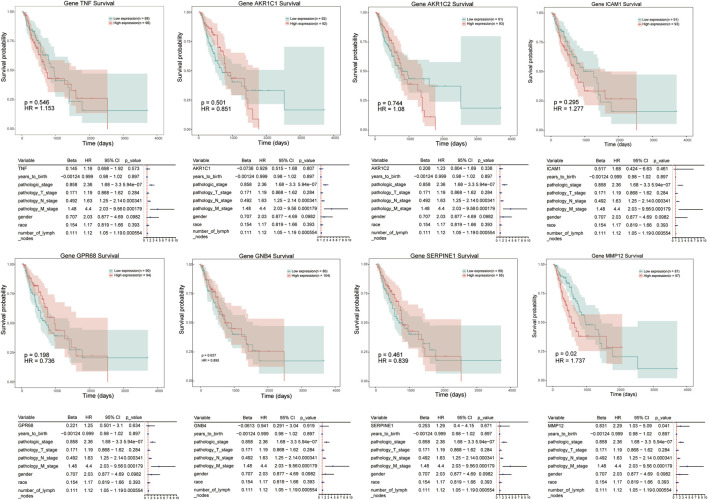
Kaplan–Meier survival curves of candidate genes in ESCC, including TNF, AKR1C1, ICAM1, MMP12, et al. Red line represents high expression of crucial genes while green line refers to low expression genes. The *X* axis represent overall survival time (day), and *Y* axis means survival probability.

We conducted a survival analysis on independent cohort of patients (the data were derived from TCGA database) to analyze the prognostic significance of 8 genes in ESCC patients. The analyzed signatures included genes TNF, AKR1C1, AKR1C2, and ICAM1, et al. (Figure 5) The patients were divided into high expression group and low expression group according to the median of gene expression. Survival probability was compared between two groups to predict candidate genes with prognostic value. The results revealed that patients with high level of MMP-12 exhibited poorer prognosis than patients in low expression groups (hazardous ratio for survival probability, 1.737; *p* < 0.05).

**FIGURE 6:**
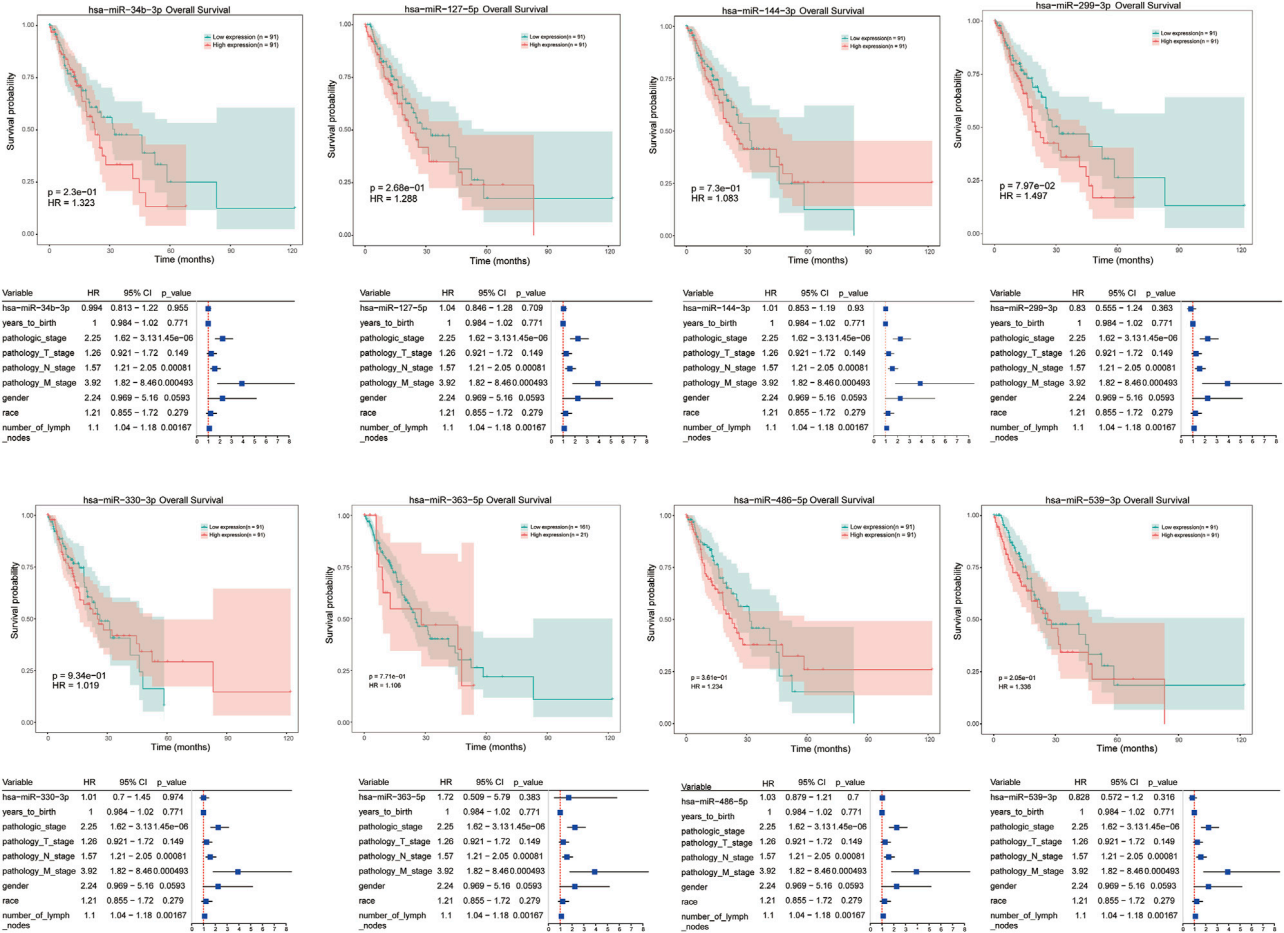
Univariate cox regression analysis and survival analysis for differentially expressed miRNAs in ESCC responder samples, including hsa-miR-34b-3p, hsa-miR-127-5p, hsa-miR-144-3p, etc. The X axis represent overall survival time (month), and Y axis means survival probability. *p* < 0.05 represent a significant difference.

Moreover, univariate cox regression analysis and survival analysis were performed for these differentially expressed miRNAs, such as hsa-miR-34b-3p, hsa-miR-127-5p, hsa-miR-144-3p, and hsa-miR-486-5p et al. However, the results revealed that there was no significant correlation between any dysregulated miRNAs and prognosis of ESCC patients (Figure 6).

## Discussion

In this study, we attempted to identify potential tumor biomarkers for prediction of nCRT-response in patients with ESCC. After integrating overlapped genes between RNA sequencing dataset and GEO datasets, we identified 17 genes that were upregulated, and eight genes that were downregulated in these two datasets. After verification of the data set samples used in the project analysis and the actual number of samples included in the analysis, no samples were excluded from the project, and all samples were analyzed. Functional enrichment analysis showed that dysregulated genes were mainly involved in “response to stimulus”, “regulation of signal transduction”, “AGE-RAGE signaling pathway in diabetic complications”, “steroid hormone biosynthesis”. According to analysis of PPI network and miRNA-mRNA network, we identified eight candidate genes, TNF, AKR1C1, AKR1C2, ICAM1, GPR68, GNB4, SERPINE1 and MMP12, that were associated with progression of ESCC progression. Finally, univariate regression analysis in ESCC patients revealed that high expression of MMP12 was significantly correlated to poor prognosis.

Previous studies have explored the difference of gene expression between nCRT responder and non-responder samples for prediction of nCRT response in esophageal cancer. Maher et al. (2009) identified eight genes that were differentially expressed in patients responded to treatment. Based on analysis using predictive model, five genes were able to predict response to nCRT with high accuracy (95%) in a large proportion of esophageal cancer patients. In a recent study (Chen et al., 2015), researchers identified nine dysregulated genes between treatment responders and non-responders. Functional enrichment analysis showed that four of the genes, miR-422, CDK4, Cyclin D2, and E2F3, were mainly related to G1/S checkpoint which could regulate tumor sensitivity to nCRT. Els Visser et al. (2017) assessed the association of gene expression and clinical outcome in 22 studies of esophageal cancer, and they found a large heterogeneity in gene expression, response to nCRT, and lymph node metastasis.

Our results demonstrated that eight genes, including TNF, AKR1C1, AKR1C2, ICAM1, GPR68, GNB4, SERPINE1 and MMP12, were candidate genes associated with ESCC progression. AKR1C1/C2 encodes enzymes belong to aldo/keto reductase superfamily. These enzymes are crucial in drug resistance of cancer cells and related to metabolism of polycyclic aromatic hydrocarbons (Selga et al., 2008). AKR1C1 and AKR1C2 share a high degree of homology in AKR1C subfamily and were different in seven amino acids. Previous study showed that AKR1C1/C2 were associated with EDHB-induced inhibition of esophageal cancer cell proliferation and this might promote treatment of esophageal cancer using EDHB, a substrate of the enzymes (Li et al., 2016).

In the miRNA-mRNA regulatory network, we identified PER2, PCTP, TNF and hsa-miR-486-5p as hub genes related to disease progression. MiRNA have been confirmed to have a major role in cancer development by regulating the expression of oncogenes or cancer suppressor genes. Aberrant expression of special miRNAs have been detected in ESCC patients such as upregulated of miR-10b (Tian et al., 2010), miR-21 (Mathe et al., 2009), miR-26a (Shao et al., 2016) and downregulated miR-125b, miR-203, and miR-205 (Matsushima et al., 2011; Hu et al., 2016; Fan et al., 2018). A recent study showed that downregulation of miR-486-5p was reported in esophageal carcinoma samples and it might function as a tumor suppressor gene in disease metastasis via regulating cellular migration (Yi et al., 2016). By tissue microarrays analysis of 185 ESCCs samples, Ren et al. also found that decreased miR-486-5p was identified in 66.2% of cases and abnormal expression of miR-486-5p were related to prognosis of esophageal carcinoma (Ren et al., 2016). Our results predicted down-regulated miR-486-5p, interact with target gene ICAM1 played critical roles in ESCC progression. ICAM1 or CD54 is a 90 kDa glycosylated transmembrane protein highly expressed on endothelial cells and mesenchymal stem cells. It plays major roles in cell metastasis, proliferation and multiple cellular immune response. Dysregulation of ICAM1 has been confirmed in liver cancer and ESCC stem cells (Liu et al., 2013; Tsai et al., 2015). ICAM1 promotes epithelial-to-mesenchymal transition by regulating metastasis-related genes in ESCC cells. ICAM1 was identified as a target gene of lncRNA-ECM and involved in development and progression of ESCC (Yao et al., 2018). However, the role of ICAM1 and miR-486-5p remains unclear, especially the interaction of the two molecules. A previous study reported that down-regulation of miR-486-5p could suppress tumor metastasis by regulating metastatic mediator of ICAM-1 in breast cancer (Abdallah et al., 2017). It is speculated that ICAM-1 might be regulated by miR-486-5p and played a crucial role in development and metastasis of ESCC. Therefore, inhibition of ICAM-1 might be a potential strategy for ESCC treatment.

In addition to hsa-miR-486-5p, survival analysis was performed for several other differentially expressed miRNAs, such as hsa-miR-34b-3p, hsa-miR-127-5p, and hsa-miR-144-3p, et al. in tumor samples. However, there was no significant correlation between any dysregulated miRNAs and prognosis of ESCC patients. According to literature, miR‐127 was downregulated in several types of cancers, including gastric cancer (Guo et al., 2013), glioblastoma (Jiang et al., 2014), and hepatocellular carcinoma (Huan et al., 2016). In ESCC patients, Gao et al. revealed that miR-127 acted as a tumor suppressor in tumors by regulating oncogene Formin-like 3 (FMNL3) (Gao et al., 2016). In another study, seven serum miRNAs were identified as ESCC biomarkers, including miR-127-3p (Zhang et al., 2010). Also, tissue or serum miR-144 expression were evaluated in gastric cancer and low miR-144 expression was found to predict a poor prognosis in gastrointestinal cancer (Liu et al., 2017). Moreover, miRNA-34b played an oncogenic role in ESCC development, the polymorphisms of rs4938723/pri-miR-34b/c were associated with ESCC susceptibility based an analysis on a large number of Chinese population (Harata et al., 2010; Zhang et al., 2014). Thus, the potential role of these miRNAs in response to nCRT in ESCC warrants further studied.

Protein of MMP-12 is known as human macrophage metalloelastase (HME) or macrophage elastase (ME), and belongs to the MMPs family. MMP12 is associated with elastin degradation and macrophage migration in various diseases, such as chronic obstructive pulmonary disease, skin diseases and cancers (Kerkela et al., 2000; Hunninghake et al., 2009). However, the function of MMP12 in tumors is controversial. Aberrant expression of MMP12 was reported in several types of cancers, including hepatocellular carcinoma (He et al., 2018), lung cancer (Roman, 2017), colon cancer (Klupp et al., 2016) and nasopharyngeal carcinoma (Chung et al., 2014). Overexpression of HME/MMP12 mRNA in patients with colorectal carcinoma exhibited a significantly better survival outcome compared with patients with normal HME/MMP12 mRNA expression (Yang et al., 2001). Cheng et al. observed a high level of MMP12/HME protein in patients with gastric carcinoma, and overexpression of MMP12 represented a better survival rate (Cheng et al., 2010). The anti-tumorigenic effect of MMP12 might be due to the generation of angiostatin, which is induced by MMP12 and could prevent tumor angiogenesis. However, in other types of cancers, upregulated MMP12 were reported to involve in short survival times. When grouping samples, the analysis is based on the average gene expression to distinguish high and low expression, so the sample size of high and low expression is different. The longest survival time of high expression group is around 2000 days, which is also consistent with the survival trend of grouping according to the median. To our knowledge, the potential role of MMP12 in ESCC remains uncertain. A recent study showed that overexpression of MMP12 was identified in cohorts of resectable tumor tissues compared with normal squamous epithelium, and overexpression of MMP12 was correlated with poor overall survival in ESCCs (Han et al., 2017). Consistent with the previous study, our results also demonstrated that overexpression of MMP12 was correlated with clinical stage and poor survival outcomes by cox regression analysis. In miRNA-mRNA regulatory network, MMP12 interacting with TNF was identified as hub genes related to disease progression. A previous study have reported that the expression and secretion of MMP-12 can be regulated by IL-1β and TNF-α in human airway smooth muscle cells, and thereby participated in diseases of the airway, such as chronic asthma or chronic obstructive pulmonary diseases (COPD) (Xie et al., 2005). Yu et al. showed that TNFα-activated mesenchymal stromal cells can recruit CXCR2+ neutrophils to tumor microenvironment, resulting in upregulation of MMP12 and other metastasis-related genes in tumor cells, such as MMP13 and TGFβ, that promoted breast cancer metastasis (Yu et al., 2017). However, there is few study on the interactions of MMP12 and TNFs in cancer development. Our findings suggested that MMP12 might interact with TNFs and play a role in ESCC progression.

There were some limitations in our study. Experimental validation should be conducted to identify the exact biological behaviors of candidate DEGs or miRNAs in ESCC development. Meanwhile, the number of ESCC specimen was limited. Further validation in larger cohorts is necessary to investigate the disease predictive value of these genes.

## Conclusion

In conclusion, we identified eight genes, including TNF, AKR1C1, AKR1C2, ICAM1, GPR68, GNB4, SERPINE1, and MMP12, as candidate genes by performing integrative analysis on gene expression profiles of microarray datasets. We found that abnormal expression of MMP12 was significantly correlated with pathological degree, TNM stage, lymph nodes metastasis, survival time of ESCC patients. Further basic experiments and large-scale multi-center clinical research studies are required to validate our results since our study was conducted based on data analysis.

## Data Availability

The datasets presented in this study can be found in online repositories. The names of the repository/repositories and accession number can be found below: https://www.ncbi.nlm.nih.gov/,GSE137867.

## Abbreviations

GO, gene ontology; KEGG, Kyoto encyclopedia of genes and genomes; OS, overall survival; BP, biological process; CC, cellular component; ESCC, esophageal squamous cell carcinomas; GEO, gene expression omnibus; DEGs, differentially expressed genes; nCRT, Neo-chemoradiotherapy; PPI, protein-protein interaction network; log2 FC, log2 fold change/logarithm of fold change.
